# Legionellosis Caused by Non-*Legionella pneumophila* Species, with a Focus on *Legionella longbeachae*

**DOI:** 10.3390/microorganisms9020291

**Published:** 2021-01-31

**Authors:** Stephen T. Chambers, Sandy Slow, Amy Scott-Thomas, David R. Murdoch

**Affiliations:** Department of Pathology and Biomedical Science, University of Otago, Christchurch 8011, New Zealand; Sandy.slow@otago.ac.nz (S.S.); Amy.scott-thomas@otago.ac.nz (A.S.-T.); David.murdoch@otago.ac.nz (D.R.M.)

**Keywords:** *L. longbeachae*, *Legionella*, epidemiology, environment, pathogenesis, prevention

## Abstract

Although known as causes of community-acquired pneumonia and Pontiac fever, the global burden of infection caused by *Legionella* species other than *Legionella pneumophila* is under-recognised. Non-*L. pneumophila* legionellae have a worldwide distribution, although common testing strategies for legionellosis favour detection of *L. pneumophila* over other *Legionella* species, leading to an inherent diagnostic bias and under-detection of cases. When systematically tested for in Australia and New Zealand, *L. longbeachae* was shown to be a leading cause of community-acquired pneumonia. Exposure to potting soils and compost is a particular risk for infection from *L. longbeachae*, and *L. longbeachae* may be better adapted to soil and composting plant material than other *Legionella* species. It is possible that the high rate of *L. longbeachae* reported in Australia and New Zealand is related to the composition of commercial potting soils which, unlike European products, contain pine bark and sawdust. Genetic studies have demonstrated that the *Legionella* genomes are highly plastic, with areas of the chromosome showing high levels of recombination as well as horizontal gene transfer both within and between species via plasmids. This, combined with various secretion systems and extensive effector repertoires that enable the bacterium to hijack host cell functions and resources, is instrumental in shaping its pathogenesis, survival and growth. Prevention of legionellosis is hampered by surveillance systems that are compromised by ascertainment bias, which limits commitment to an effective public health response. Current prevention strategies in Australia and New Zealand are directed at individual gardeners who use potting soils and compost. This consists of advice to avoid aerosols generated by the use of potting soils and use masks and gloves, but there is little evidence that this is effective. There is a need to better understand the epidemiology of *L. longbeachae* and other *Legionella* species in order to develop effective treatment and preventative strategies globally.

## 1. Introduction

Following the discovery of *Legionella pneumophila* as the cause of the Legionnaires’ disease outbreak in Philadelphia in 1976 [1], it was quickly realised that there were other species within the genus *Legionella*, some of which could also cause human disease. Nearly 90 different *Legionella* species have now been reported, of which more than 20 different species are known to be pathogenic in humans [2,3]. A list of species currently known to cause human disease is available online [4]

Despite recognition that a variety of *Legionella* species and serogroups can cause human disease, *L. pneumophila* is overwhelmingly reported as the most common cause of legionellosis globally. While the predominance of *L. pneumophila* may be a true observation, there is inherent diagnostic bias in current testing strategies for legionellosis that favours detection of *L. pneumophila* over other *Legionella* species. In particular, many countries rely on the urinary antigen test in cases of suspected Legionnaires’ disease. This test only reliably detects *L. pneumophila* serogroup 1, so other *Legionella* species and serogroups may not be tested for at all if this is used as the sole, or predominant testing method. As a consequence, detection of infections caused by *Legionella* species other than *L. pneumophila* are usually made by chance, and the true burden and epidemiology of disease is largely unknown. This situation is further complicated by the lack of studies on the microbial aetiology of community-acquired pneumonia that systematically test for all species of *Legionella* using PCR and/or culture-based methods.

**Figure 1:**
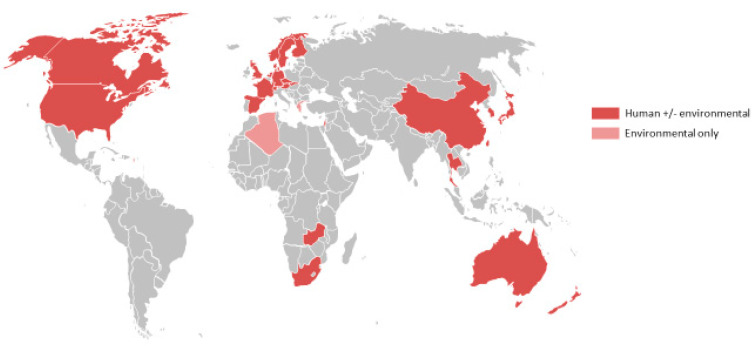
Reported global distribution of *L. longbeachae* isolated from human and/or environmental sources [5,8,9,10,11,12,13,14,15,16,17,18,19,20,21,22,23,24,25,26,27,28,29]. Data from Austria, Norway and Slovakia provided by Julien Beauté, European Centre for Disease Prevention and Control (ECDC); and for the Czech Republic by Vladimir Drašar, Head Public Health Institute Ostrava, National Legionella Reference Laboratory, Czech Republic.

The one exception is *Legionella longbeachae*, which was first isolated from patients with pneumonia in the USA 40 years ago [5]. In some countries, such as Australia and New Zealand, where the aetiology of community-acquired pneumonia has been systematically studied, *L. longbeachae* disease has emerged as the predominant cause of legionellosis [6,7]. Cases are increasingly being reported from other regions as well. The countries that have reported at least one case are shown in Figure 1, demonstrating that the conditions for infection may occur in countries with widely varying climates and socioeconomic conditions. However, it is still unclear whether the relatively high incidence of Legionnaires’ disease caused by species other than *L. pneumophila* in Australia and New Zealand is a true geographic variation or simply reflects that these species are more rigorously tested for in this region.

This paper reviews legionellosis caused by *Legionella* species other than *L. pneumophila.* The primary focus is on legionellosis caused by *L. longbeachae*, simply because considerably more data are available on this species. Indeed, apart from *L. pneumophila*, *L. longbeachae* is the only species causing legionellosis for which there are sufficient data to make detailed descriptions about clinical features and epidemiology.

## 2. Clinical Features

The clinical spectrum of legionellosis caused by *Legionella* species other than *L. pneumophila* is similar to that caused by *L. pneumophila*, with pneumonia (Legionnaires’ disease) being the predominant clinical presentation. There are no clinical features that distinguish Legionnaires’ disease caused by *L. pneumophila* from *L. longbeachae*, nor can Legionnaires’ disease be it distinguished clinically from other causes of community-acquired pneumonia [30,31]. Increasing age, immune suppression and pre-existing lung disease have been regarded as important predisposing conditions for legionellosis. Recognition of these factors has led some researchers to develop scoring systems to increase recognition of Legionnaires’ disease, but none of these have been developed for non-*L. pneumophila* species or gained widespread acceptance as a clinical tool [32]. A case series of *L. longbeachae* disease from New Zealand found that a higher proportion of cases were males (63%), most cases (59%) were of mild to moderate severity (CURB-65 score 0–1), 25% required admission to the intensive care unit, and the mortality rate within 30 days of admission was 5% [31].

Pontiac fever, is transient flu-like illness with a high attack rate, [33] and has been associated with exposure to a variety of *Legionella* species, including *L. pneumophila*, *Legionella feeleii*, *Legionella micdadei*, *Legionella anisa*, and *L. longbeachae* [34,35,36,37,38,39]. Outbreaks of Pontiac fever caused by species other than *L. pneumophila* have occurred in a variety of occupational and recreational settings, including workers in an automobile manufacturing plant (*L. feeleii*) [34], users of whirlpool spas (*L. micdadei*) [35,36,40], workers at a compost manufacturing site (*L. longbeachae*) [37] and conference delegates and restaurant patrons exposed to decorative fountains (*L. anisa*) [38,39]. Reports of Pontiac fever are almost certainly underestimated because sporadic cases are unlikely to come to attention and be diagnosed. 

Non-*L. pneumophila* has also been identified in cases of skin and soft tissue infection, septic arthritis and bacterial endocarditis [41,42,43,44,45,46,47,48,49,50]. These cases may be severe and difficult to diagnose as legionellae are unexpected as the infecting organism. A recent example is that of a 78-year-old gardener who pricked her hand on a rose thorn. *L. longbeachae* was cultured on selective media from surgically debrided tissue that would have been missed with standard investigations [41]. Likewise, diagnosis and treatment of recurrent upper limb abscesses of *L. bozemanii* was delayed for weeks in a patient on steroid therapy and methotrexate for seronegative arthritis; the diagnosis was eventually made by 16S rRNA PCR and seroconversion [51].

## 3. Epidemiology

The epidemiology of infection with *L. longbeachae* and other non-*L. pneumophila* species of *Legionella* is poorly described worldwide, although cases have been reported in many countries (Figure 1). A major reason for this is the difficulty with diagnosis, with the likelihood that cases are markedly under-diagnosed and under-recognised. In many centres, testing is primarily based around urinary antigen testing that recognises only *L. pneumophila* serogroup 1. Culture of respiratory samples is discouraged by some authorities for nonsevere disease, including in hospitalised patients [52]. The results may also be compromised if specimens are taken after empiric therapy (including antilegionella therapy) has been administered. Where other techniques are employed, such as serology, specific culture techniques and PCR, *L. longbeachae* has emerged as an important cause of Legionnaires’ disease in community-acquired pneumonia. Other non-*L. pneumophila* species are recognised particularly in severely immunocompromised patients [20,53,54]. The non-*L. pneumophila* species almost certainly cause many more cases of mild to moderate disease that do not come to medical attention and remain an ongoing burden of morbidity on the population.

### 3.1. Incidence

Several high-income countries and regions have well established surveillance systems but almost all data on legionellosis relies on various forms of passive surveillance (Table 1). Most of these data are derived from cohort studies or case reports of patients who required admission to hospital. These reports demonstrate striking differences in the number of cases identified in Australia and New Zealand compared with Europe and North America [6,55,56,57,58]. Most cases are sporadic, although clusters of Legionnaires’ disease caused by *L. longbeachae* have been recognised in Scotland, where six cases of Legionnaires’ disease caused by *L. longbeachae* were identified over a four-week period in 2013 [59], and in Sweden, where 30 cases were identified during spring/summer 2018 [60].

The highest reported incidence rates of Legionnaires’ disease from any cause and from *L. longbeachae* have been reported from New Zealand. Since 1999 in the Canterbury region, routine passive surveillance, based on the culture of respiratory samples, demonstrated that *L. longbeachae* was a major cause of Legionnaires’ disease in New Zealand. To increase the sensitivity of the testing methods, PCR was adopted as the primary diagnostic tool for all species of *Legionella* in respiratory samples submitted to the diagnostic laboratory in 2010. This technique demonstrated there was a large number of cases that were previously unsuspected, and there was a marked seasonal variation in cases with the peak occurring in spring and early summer [53]. Because there were differences in diagnostic practices around New Zealand, a nationwide study was conducted in 2015–6 using the same method of recruitment to properly assess the nationwide burden [7]. This gave an annualized rate of 5.4 per 100,000 people per year. *L. longbeachae* was the predominant species identified and was implicated in 150 (63%) of 238 Legionnaires’ disease cases. This is the highest rate of Legionnaires’ disease reported caused by both *L. longbeachae* and *L. pneumophila* worldwide.

A major limitation of this study was the reliance on testing only those samples submitted to the laboratory. A small study using enhanced sputum collection of hospitalised patients with community-acquired pneumonia found >40% of pneumonia patients in our study were unable to spontaneously expectorate sputum, but this could be significantly increased to 67% by extra assistance or sputum induction [62]. This increased the case detection of Legionnaires’ disease by 36%. Some cases were almost certainly missed in this study as a dry cough is more common in Legionnaires’ disease than other causes of community-acquired pneumonia [31].

In Australia over the period of 2009 to 2015, the annual notification rate of Legionellosis using passive surveillance was about 1.5 per 100,000 people [6]. *L. longbeachae* was the more commonly identified (144 to 215 notified cases) compared with *L. pneumophila* (114 to 228 cases). *L. longbeachae* was most common (>50%) in the Mediterranean climate of Western Australia and the tropical Northern Territories.

In contrast, *L. longbeachae* was reported in low numbers elsewhere, making up 0.3–3.6% of cases in the US, 0.7% in Japan, and 1% in Europe (Table 1). There is some preliminary evidence that cases of *L. longbeachae* are increasing in both Scotland [63] and the Netherlands [64]. There is very little information on the epidemiology of Legionnaires’ disease from low and middle-income countries, but a year-long population-based study of the microbiological causes of clinically defined community-acquired pneumonia requiring hospitalisation in a rural province of Thailand has been reported [27]. Infection with *L. longbeachae* was identified by seroconversion using an indirect immunofluorescence assay of combined IgG, IgA and IgM titres. These cases were not confirmed by culture or PCR testing. The incidence rate was 5–29 cases per 100,000 of the population, incidence increased with age, and it was more common among males (rate ratio 1.6 95% CI 1.1–2.3). There was also a single case of Legionnaires’ disease caused by *L longbeachae* reported in a child in Zambia [29]. This was the only case found as part of a year-long study conducted in five locations in Africa and two in Asia of 2757 cases of childhood community-acquired pneumonia.

The epidemiologic evidence of human infection of other non-*L. pneumophila* species is limited. These infections are rarely reported due, at least in part, to diagnostic bias that precludes cases being identified. There appears to be little difference in the distribution or reported incidence rates of these species across the world. Of these, *L. micdadei* is the most consistently reported across regions (Table 1). Clusters of cases related to a common source have been described, including 12 cases of Legionnaires’ disease caused by *L. micdadei* among 38 renal and cardiac transplant patients in one centre over a three-month period, and further cases were detected 16 months later [65]. Subsequently, 16 cases of *L. micdadei* were reported in another tertiary care centre over a 12 year period, predominantly among immunocompromised hospitalised patients [66]. Another cluster of five cases of nosocomial pneumonia caused by *L. bozemanii* in a community hospital among immune compromised patients has been identified [67]. Case series have demonstrated that Legionnaires’ disease may be caused by a wide variety of non-*L. pneumophila* species in immune-compromised patients. The MD Anderson Centre in Houston Texas reported consecutive cases of *Legionella* infections in cancer patients from 2002 to 2014. Most of the 33 strains identified were on specimens obtained at bronchoscopy, and 18 were non-*L. pneumophila* species [54]. Twenty-seven patients had underlying hematologic malignancies and 23 were leukopenic. Six patients were recipients of allogeneic hematopoietic stem cell transplant, with their infections caused by five *Legionella* species. Legionnaires’ disease was the immediate cause of death in six [54]. Some cases may be related to travel but the numbers are difficult to obtain as the species of infecting *Legionella* species may not be reported [68,69].

### 3.2. Environmental Studies

#### 3.2.1. Potting Soils and Gardens

An important epidemiological link has been made between exposure to compost or potting soils and *L. longbeachae* infection. An investigation during 1988–1989, into 22 cases of *L. longbeachae* infection in South Australia, found cases were among regular gardeners. *L. longbeachae* was subsequently isolated from compost and potting soils found at the homes of four cases, providing a possible link to a natural habitat for this bacterium [70,71]. Further studies have shown that *L. longbeachae* infection was linked to the use of potting soils or gardens in other states in Australia, as well as Japan, the United States, Canada, the Netherlands and Scotland through case-series and laboratory evidence [59,64,72,73,74,75,76]. In the Scottish cluster all cases were associated with amateur gardeners and required admission to the intensive care unit [59]. A case control study in South Australia found recent use of potting mix was associated with illness (OR 4·74, 95% CI 1·65–13·55, *p* = 0·004) [72]. Similarly, exposure to purchased compost was a risk factor for Legionnaires’ disease from *L. longbeachae* in New Zealand (OR 6.2, 95% CI 2.2–17.3), unlike for homemade compost [77].

Seminal studies by Steele et al. further implicated potting soils as a source of human *L. longbeachae* infection. *L. longbeachae* was recovered on selective media from soil samples and potting mixes from four patients who suffered Legionnaires’ disease with this organism. The bacteria were able to persist for seven months in potting soil held at room temperature but did not grow at 43°C or above in soil [70]. Subsequently *L. longbeachae* was isolated from 26 (56%) of 45 potting mixes manufactured in Australia but not from imported potting soils [78]. *L. longbeachae* was found in one of four samples of fresh pine sawdust and in a soil sample close to the site, but not hammer milled bark. *L. longbeachae* was also found at two of six large scale manufacturing plants and in samples from three of 20 home compost heaps [78]. *L. Longbeachae* has since been detected in potting soils and gardens from many other countries in Europe, North America and Japan, but not as frequently as in Australia [16,79,80,81,82,83,84]. There is a highly diverse pool of *L. longbeachae* genotypes within the samples from potting soils that makes it difficult to identify the source of a clinical infection [85].

Steele and colleagues also demonstrated that the ecology of *Legionella* species in potting soils was extremely complex, as multiple species of pathogenic *Legionella* were usually present in Australia-made potting soils [86]. Among 20 Australian potting soils, *L. anisa, L. bozemanii, L. micdadei, L. gormani*, *L. dumoffi* and *L. pneumophila* serogroups 1–14 were found, in addition to *L. longbeachae* [78]. A similar range of *Legionella* species were found in 20 home composts, but with a lower proportion of *L. longbeachae* present among the 29 isolated (*L. pneumophila* serogroup 2–14, 11 strains (55%); *L. pneumophila* SG, 1 strain (5%); *L. anisa*, 5 strains (25%); *L. longbeachae* and *L. bozemani*, three strains each (15%); unidentified, six (30%)) [86]. UK studies of 24 compost products found 15 (62.5%) samples tested contained *Legionella* species either on first testing or after enrichment by coculture with amoeba when retested 8–10 weeks later. The most common species identified before enrichment was *L. sainthelensis* (5/24; 21%), while *L. longbeachae* (4/24; 17%) was the most common after amoebal enrichment. Other identified species known to cause human disease were also isolated (*L. anisa, L. bozemanii, L. micdadei, L. gormani*, *L. dumoffi, L. birminghamensis* and *L. pneumophila*) [73,83]. A similar range of *Legionella* species have been identified in potting soils in Greece, Switzerland and Japan [16,79,82]. These results suggest that potting soils could be a source of other *Legionella* infections, including with *L. pneumophila* species.

*L. longbeachae* and other non-*L. pneumophila* species have been found in water contaminated by soil, including rain water puddles in Europe, and to be widely distributed among fresh water and marine waters in Puerto Rico [87,88]. *Legionella* species were also found in water collected in rain forests from epiphytes 30 feet above ground [87]. *L. longbeachae* was not found in soil or other environmental samples obtained from around houses and work places of people in Southern Thailand where a high prevalence of *L. longbeachae* infection had been found by serological testing [89]. Many other *Legionella* species were found, suggesting one or more of these may have been responsible for the infections.

One possible difference of European potting mixes compared to and Australian and New Zealand products is the range and proportions of the constituents used in the manufacturing process. Australian and New Zealand potting soils contain a significant amount of partially or fully composted pine bark and sawdust in addition to peat, and composted green waste. Recent studies in New Zealand using qPCR identified *L. longbeachae* DNA in pine bark, pine sawdust and potting soil products containing pine products, but did not identify it in peat or commercially composted green waste [90]. Studies from Europe indicate that *Legionella* species, including *L. pneumophila* and *L. bozemanii* are common in composted green waste, but not fresh green waste. *L. longbeachae* is rarely found in either fresh or composted green waste [80]. Australian, New Zealand and European studies have failed to find *Legionella* species in peat. These studies raise the possibility that composting facilities are contaminated with *Legionella* species from other sites [78,83,90]. Studies of bark from living pine trees in New Zealand found *L. longbeachae* DNA is common in spring, but very uncommon on bark from other tree species [91]. *L. longbeachae* could be brought into composting facilities with bark, and the load may contribute to the seasonal variation in clinical cases. In Switzerland, it has been suggested that *Legionella* species could be brought into the storage facilities by wind and rain [79].

All of these studies indicate that composted material is an important reservoir of pathogenic species of *Legionella* and are a potential source of human infection, but we are unaware of any substantial data linking cases of Legionnaires’ disease to the manufacture of compost. There is some evidence that compost facilities can release bioaerosols containing *Legionella* species, and transmission of *L. longbeachae* aerosol has been suggested as a cause of a nosocomial infection [92,93].

#### 3.2.2. Water Bodies

There have been occasional reports of nosocomial *L. longbeachae* Legionnaires’ disease associated with detection of this species in hospital and residential water supplies and unpasteurised water tanks [94,95]. *L. longbeachae* has been identified in samples taken from a cooling tower, but infection from this source was not proven [96]. It has also been isolated from a hot spring water in Algeria by culture and molecular methods [8].

In contrast to *L. longbeachae*, infection with other non-*L. pneumophila* species have typically been associated with water bodies, and the epidemiology of water related non-*L. pneumophila* species is likely to be similar to *L. pneumophila.* The environmental sources of community acquired Legionnaires’ disease has been extensively reviewed [97]. Nosocomial Legionnaires’ disease with *L. micdadei, L. bosemani, L. feelii*, and *L. anisa* has been attributed to infected water distribution systems, including those in hospitals [58,98,99,100]. *L. micdadei* has also been identified in ultrasonic nebulisers [101]. *L. micdadei* has been found in whirlpool spas in Norway, and *L. anisa* associated with a decorative fountain [35].

## 4. Pathogenesis

The pathogenesis of *Legionella* infection has been most extensively studied for *L. pneumophila*, and this has been comprehensively reviewed [102]. Relatively recent advances in high throughput next generation sequencing technologies has allowed the genomes of non-*L. pneumophila* species to be sequenced and examined in greater depth. As such, comparative genomic analysis has proven to be instrumental in elucidating the factors that are important for the pathogenicity of *Legionella* species. This has revealed both important parallels, and differences between the various pathogenic factors of *Legionella* species, which remain under continued investigation.

Analysis of the genomes of up to 58 different *Legionella* species revealed they are highly dynamic and diverse, with lengths ranging in size from the smallest at 2.37 Mb in *Legionella adelaidensis* to the largest at 4.82 Mb in *Legionella santicrucis* [103,104]. Indeed, across the *Legionella* pan genome only 6% of the genes (1008) constitute the core genome [103,104], illustrating the high genomic diversity and the importance of horizontal gene transfer and recombination in shaping the genome of the genus. At around 4.1 Mbp, the single circular chromosome of *L. longbeachae* is approximately 500 kb larger than *L. pneumophila*, but has a similar GC content at around 37% [104,105,106]. The gene organization is also markedly different to *L. pneumophila*, with limited gene synteny, although there are small clusters where the gene order is highly conserved [105,106].

There are around 3500 predicted coding sequences in the *L. longbeachae* genome, and around 2000 of these have been assigned putative functions [105,106,107,108,109]. Only 65% of the genes in *L. longbeachae* are orthologous to those in *L. pneumophila* [105,110]. The *L. longbeachae* genome, as a result of its soil and plant habitat, contains numerous genes that reflect its ecological niche, with several genes that encode proteins that can degrade components of plant cell walls, such as cellulases, hemicellulases and pectin lyases [105]. In addition, the genome is highly methylated with m^4^ C and m^6^ A epigenetic modifications throughout the genome, where m^6^ A is strongly associated with particular sequence motifs [109]. Although the *L. longbeachae* genome appears to be more stable than that of *L. pneumophila* [105], large-scale rearrangements have occurred in some regions of the chromosome with evidence of extensive recombination [85].

In the accessory genome, *L. longbeachae* strains NSW150 and D4968 [106] were both found to contain a similar plasmid of around 70 kb. It was first thought that *L. longbeachae* plasmids only circulated within this species and were distinct from those found in *L. pneumophila*, which range in size from around 59 kb to 131 kb [110]. More recently, sequencing data from multiple clinical and environmental *L. longbeachae* isolates has shown that most contain at least one plasmid ranging in size from around 70 kb to 150 kb [85,109]. Despite still relatively limited data, the plasmids have been shown to have a common backbone including conjugational genes separated by variable regions. Importantly, there is evidence of extensive recombination and horizontal gene transfer between the plasmids from different species, indicating both intra and interspecies exchange of genetic material across the genus [85,107,108,109].

Transcriptome analysis of *L. pneumophila* and *L. longbeachae* has shown that both have a biphasic lifestyle (a replicative/avirulent phase and a transmissive/virulent phase), although this is less pronounced in *L. longbeachae* [105]. Both also typically exist in biofilm forms or intracellularly with amoeba or macrophages. Conditions which disrupt the biofilm may release large numbers of organisms into the environment [111]. Likewise, the ability to multiply within free-living amoeba may facilitate transmission, as a single amoebal cyst may contain thousands of *Legionella* bacteria [93]. The major route of infection is probably by inhalation of aerosols derived from soil or water, but may also reach the lung by microaspiration [112]. The infectious dose has not been precisely defined and may be higher than 1000 organisms [113]. This is almost certainly lower in people with pre-existing lung disease that disrupts the mucociliary escalator, allowing the bacterium to reach the alveolar space. Immune deficiencies, particularly cell mediated deficiencies, such as haematological malignancy, as well as immunosuppressive therapy such as dose steroid therapy, tumor necrosis factor inhibitors and anti CD-52 agents, are also important risk factors for developing disease from *Legionella* species [114,115,116]. Mucosal associated invariant T (MAIT) cells that are resident in the lung, also appear to contribute to protection against fatal infection with *Legionella*. The mechanism is dependent on the major histocompatibility complex (MHC) class I-related molecule MR1, interferon-γ (IFN-γ) and granulocyte macrophage-colony stimulating factor (GM-CSF) [117].

On reaching the alveolar space *L. pneumophila* is known to attach to alveolar macrophages using flagella, pili and outer membrane porins [118,119,120,121,122,123]. The attachment enables phagocytosis to follow, mediated by macrophage infectivity potentiator protein (MIP) and human complement C3. In contrast *L. longbeachae* lack flagella but have other effector proteins, possibly derived from organisms that typically live in soil and plant derived material. The lack of flagella has been cited as one of the reasons for the different responses to infection in different strains of mice, where A/J mice are susceptible to *L. pneumophila* infection and replication, but C57BL/6 and BALB/c mice are not [110,124]. In contrast, all three mouse strains are susceptible to *L. longbeachae* infection and replication. It is thought that the flagellin of *L. pneumophila* triggers pyroptosis and cell death in the C57BL/6 and BALB/c mice, leading to a failure to establish infection [110,124].

Following phagocytosis, *Legionella* infection, survival and replication within a host cell is dependent on successfully transforming the phagosome into a distinct membrane bound vacuole, termed the *Legionella* containing vacuole (LCV) [102,125]. To do this *Legionella* employ various secretion systems to translocate effector proteins into the vacuole, which, in turn, inhibit phagocyte lysosome fusion [102]. The effector proteins also facilitate the transformation of the vacuole into a nutrient rich environment, as they enable the host resources to be hijacked and transported back to the LCV to support legionella replication. As the nutrients are depleted by bacterial replication, the organism enters stationary phase, triggers apoptosis of the host cell releasing the organisms to be phagocytosed and enters a new replicative cycle.

The various secretion systems and the effector proteins of *Legionella* are the predominant drivers of their pathogenicity. Comparative genomic analysis has shown that all species analysed to date contain the genes for both a type 2 secretion system (T2SS) and a type 4B secretion system (T4BSS), also known as the Icm/Dot secretion system [102,103,126]. In addition to these, and similarly to *L. pneumophila*, *L. longbeachae* contains the genes for a type 4A secretion system (T4ASS) but, like all other non-*L. pneumophila* species, it lacks a type 1 secretion system [126]. The Icm/Dot T4BSS is crucial for LCV biosynthesis and intracellular replication. It is also the primary and most well-studied pathogenic secretion system of *Legionella* [104,125,126,127], particularly in *L. pneumophila*. The components are encoded by 25 genes that occur within two distinct pathogenicity regions on the chromosome, and the gene order and orientation is highly conserved genus-wide [104,126,127]. The Icm/Dot T4BSS translocates effector proteins into the host cell that interact with the host to subvert resources and cell function for the benefit of the bacterium and is indispensable for pathogenicity. Despite the highly conserved nature of the Icm/Dot T4BSS, the effector repertoire that it translocates into the host has been shown to be highly variable across the various species examined to date [103,104]. From species to species, the effectors differ in number and, although they share functional similarities, there is little DNA sequence homology [128]. Of the over 300 effectors identified in *L. pneumophila*, only 35% are found to occur in *L. longbeachae* [125]. Genus-wide, comparative analysis of 38 species has revealed even greater variability with only seven putative core effectors identified [104], i.e., effectors that were found to have orthologs in each of the species examined out of a total of 5885. More recently, Gomez-Valero et al., 2018, after examining 80 strains from 58 different *Legionella* species showed that the pan-genus pool of putative Icm/Dot T4BSS effectors could be well over 18,000 proteins, again illustrating the extremely high diversity of the effector proteins in *Legionella*. Furthermore, analysis of these putative effector genes revealed that they encode secreted proteins that contain a large number and variety of eukaryotic domain-encoding proteins and eukaryotic-like proteins [102,103]. The genes encoding these proteins are thought to have been acquired via horizontal gene transfer from their protozoan hosts in a type of coevolutionary arms race that allows *Legionella* to mimic the host to establish infection and successfully replicate.

The survival tactics and pathogenic trajectory of *Legionella* is significantly shaped by the interactions and exchanges that it has with its protozoan hosts, but not by interactions it has with its human host [129,130,131]. This is because human infection by *Legionella* is an evolutionary dead-end; either the bacterium dies because of successful response/treatment or the human host dies because of the infection, which consequently causes the bacterium to die as it does not have recourse to the external environment. Therefore, infectivity and pathogenicity to humans is not an evolutionary advantage to *Legionella*. While *L. pneumophila* has been found to exhibit a broad host range that spans several protozoan phyla [129], little is known about the protozoan hosts of other *Legionella* species. Nonetheless, it has been shown in various coculture studies that the protozoan host species has a major influence on *L. pneumophila’s* ability to infect human cells [132], and it is reasonable to expect that the same is true for other *Legionella* species. For instance, *L. pneumophila* cocultured in *Acathamoeba polyphaga* is more tolerant to antibiotics, while resistance to chlorination is higher in those grown in *Vermamoeba vermiformis* when compared to those from *Acathoamoeba castillanii* [132].

## 5. Prevention and Public Health

The public health aspects most specific to legionellosis caused by *Legionella* species other than *L. pneumophila* relate to *L. longbeachae* and exposure to gardening activities. The public health response to the threat posed by potting soils in New Zealand and Australia has been to place responsibility onto the individual gardener. Gardeners are alerted to the risk with warnings printed on potting soil containers and information leaflets available at the point of sale. Information on Legionnaires’ disease can be found on the Ministry of Health or State Health authorities’ websites. Gardeners, particularly those with other health conditions or advanced age, are advised to protect themselves by using gloves and masks and to dampen the potting soil when the bag is opened to minimise aerosol formation [31,70].

This advice makes intuitive sense as inhalation of aerosols is probably the most important means of transmission and is the major route of infection of *L. pneumophila*. This is supported by results of a case control study among gardeners that found that tipping or trowelling potting soils was a major risk factor for Legionnaires’ disease (population attributable fraction 65%) [77]. However, behaviours that involve touching the face, such as eating or drinking without handwashing, or smoking, may also place gardeners at risk of infection (population attributable fraction 35%) [77]. These results suggest that eliminating aerosolization activities and effective hand hygiene could reduce the risk of Legionnaires’ disease but, disappointingly, the study did not find any protective effect of wearing a mask or gloves [77].

There has been little research on the acceptability or effectiveness on the use of personal protective equipment for preventing Legionnaires’ disease while gardening. Multiple barriers to the use of face masks have been identified in other settings with serious health risks, such as tuberculosis and influenza, and contribute to poor acceptability among gardeners [133,134,135]. These include lack of perceived risk, discomfort and poor fit. Discomfort may be compounded when masks become dampened, as this increases the work of breathing and the masks subsequently lose their protective effect. Alongside discomfort, masks may also may give a false sense of security and interfere with communication, and may also accumulate bacteria and become hazardous prior to disposal [136]. It may be possible to change the behaviour of gardeners as the COVID-19 pandemic has changed public perception of the value of masks [135].

Other avenues to change gardeners’ behaviour to reduce Legionnaires’ disease risk are still to be explored. These could include warning about the risks through major media outlets during the high seasons, and physicians providing personalised information for immune compromised patients to help them prevent exposure to all *Legionella* species.

A more proactive approach in Australia and New Zealand may be to engage with the potting soil industry to develop low-risk manufactured potting soils. The apparent differences in epidemiology of Legionnaires’ disease between Europe and Australia and New Zealand suggests that there is less exposure to *L. longbeachae* in Europe. Pasteurising potting soils, or reducing or eliminating pine products from potting soils, and using peat and well composted green waste, which appear to be inhospitable to *L. longbeachae*, may reduce these infections. As peat is a limited resource, supply may become restricted for environmental reasons so that manufactures may turn to other products such as pine bark to fill the gap.

The prevention of Legionnaires’ disease in hospitals has been widely discussed, and water standards have been set by the World Health Organization and many national agencies [137,138]. These have been developed appropriately with particular reference to *L. pneumophila,* but the measures employed to minimise infection with this organism would be expected to be effective for other waterborne *Legionella* species. These include well designed hospital water systems, and maintenance of hot water systems, to ensure inhibitory temperatures are maintained. Hot water pipes need to be properly insulated to prevent the warming of adjacent cold-water pipes, stagnation sites such as blind pipe sections and holding tanks eliminated, and material that supports legionella growth minimised [139,140,141,142]. A metanalysis has shown that temperatures of 55–59°C are associated with a low probability of *Legionella* species being detectable in water samples, and in cold water kept below 20 °C [143,144].

There is a lack of consensus on the need for monitoring water systems in hospitals, with some organisations recommending against monitoring, others monitoring only those areas housing immune-compromised patients, and others all hospital water supplies [145,146]. This is in part because it has been difficult to define specific and sensitive target concentrations for remediation although recent studies have suggested methods to improve monitoring techniques [100,147,148].

If *Legionella* species are detected, methods such as the use of monochloramine, heat, ultraviolet light, copper-silver ionization and chlorination have been used to reduce the risk to patients [67,149,150,151,152,153]. Improvement of chlorination of water supplies may reduce the burden of travel-related legionellosis from non-*L pneumophila* species [151]. It is important that detection systems are not solely focused on *L. pneumophila*, as molecular techniques may help identify the source of infection. These techniques should also be used for monitoring water distribution systems that may harbour non-*L. pneumophila* species [154].

## 6. Conclusions

The number and distribution of infections caused by *L. longbeachae* and other non-*L. pneumophila* legionellae are almost certainly markedly underestimated globally. Humans are accidental hosts, where infections are most likely to occur following interactions with the natural environment. It is the relationship between the environmental conditions and the interactions *Legionella* has with is protozoan hosts that shape their pathogenesis, enabling their survival and growth. Favourable conditions are needed to allow *Legionella* numbers to increase in the environment, thereby accounting for the seasonal pattern of human infections.

Legionnaires’ disease is a preventable pneumonia. Resourcing of the public health response will inevitably be governed by the number of cases identified and how political figures assign responsibility for controlling Legionnaires’ disease. Multiple factors currently combine to mitigate against case ascertainment. These include the passive design of surveillance systems with testing of only respiratory samples submitted for analysis. Many patients with community-acquired pneumonia are not asked to produce sputum samples, and up to 40% of patients with community-acquired pneumonia are unable to expectorate sputum when asked [62]. Investigation of nonsevere cases of community-acquired pneumonia is discouraged including for hospitalised cases in many centres. This is exacerbated by a perception among some clinicians that Legionnaires’ disease is necessarily a severe infection, and only these cases are investigated, and then testing is limited to only the urinary antigen test for *L. pneumophila* serogroup 1. Compounding these issues, investigations may only be done after antimicrobial therapy has been initiated, including agents that are active against *Legionella* species, thereby reducing the sensitivity of any testing that is conducted. There may be significant numbers of cases who present to health practitioners in the community and are not investigated or are not sufficiently unwell to seek medical attention. Lastly, financial constraints discourage testing, or at least minimise the resources allocated to investigating community-acquired pneumonia.

Practice guidelines make pragmatic sense but should not override the need for well conducted studies that adequately define the causes of community-acquired pneumonia in both the general public and immune compromised patients. These studies should avoid the tyranny of urine testing only, to identify pinch points where public health interventions can minimise infection rates of non-*L. pneumophila* species.

## Figures and Tables

**Table 1:** Number of cases of Legionellosis reported from countries with national surveillance systems.

	Countries *				
*Legionella* species	New Zealand [55] (2010–2019)	Australia [6] (2014)	Europe [58] (2008–2017)	USA [61] (2016–2017)	Japan [56]
*L. pneumophila*	314 (19%)	195 (46%)	4738 (97%)	230 (60%)	679 (98%)
*L. longbeachae*	902 (54%)	164 (39%)	48 (1%)	1 (0.3%)	6 (0.1%)
*L. anisa*	3 (0.2%)	na	6 (<0.1%)	0	1 (0.1%)
*L. bozemanii*	16 (1%)	na	17 (0.3%)	2 (0.5%)	1 (0.1%)
*L. dumofii*	49 (3%)	na	6 (<0.1%)	0	1 (0.1%)
*L. feelei*	2 (0.1%)	na	1	1 (0.3%)	2 (0.3%)
*L. gormani*	9 (0.5%)	na	na	2 (0.5%)	0
*L. jordanis*	20 (1%)	na	na	0	0
*L. micdadei*	55 (3%)	2 (0.5%)	12 (0.3%)	7 (1.8%)	0
*L. sainthelensii*	29 (2%)	1 (0.2%)	1 (<0.1%)	0	0
Other	7 (0.4%)	na	8 (<0.1%)	0	2 (3%)
Not identified	85	62	25	139	0
Total cases	1684	424	4884	382	692

* Cases diagnosed by culture, urinary antigen test, seroconversion are included for all countries other than Japan, which includes culture-proven cases only. na: Not available.

## Data Availability

No new data presented.
